# CCL2 associated with CD38 expression during *ex vivo* expansion in human cord blood-derived hematopoietic stem cells

**DOI:** 10.18632/aging.203398

**Published:** 2021-08-10

**Authors:** Chao-Ling Yao, Poyin Huang, Tsai-Chi Liu, Yung-Kai Lin, Ching-Yun Chen, Yi-Ting Lai, Tzu−Yun Chin, Tsung-Yu Tseng, Yi-Chiung Hsu

**Affiliations:** 1 Department of Chemical Engineering, National Cheng Kung University, Tainan 701, Taiwan; 2 Department of Chemical Engineering and Materials Science, Yuan Ze University, Chung-Li, Taoyuan City 320, Taiwan; 3 Graduate School of Biotechnology and Bioengineering, Yuan Ze University, Chung-Li, Taoyuan City 320, Taiwan; 4 Department of Neurology, Kaohsiung Medical University Hospital, Kaohsiung Medical University, Kaohsiung City 807, Taiwan; 5 Department of Neurology, Kaohsiung Municipal Siaogang Hospital, Kaohsiung Medical University, Kaohsiung City 807, Taiwan; 6 Neuroscience Research Center, Kaohsiung Medical University, Kaohsiung City 807, Taiwan; 7 Department of Neurology, Faculty of Medicine, College of Medicine, Kaohsiung Medical University, Kaohsiung City 807, Taiwan; 8 Department of Biomedical Sciences and Engineering, National Central University, Chung-Li, Taoyuan City 320, Taiwan; 9 Institute of Food Safety and Risk Management, National Taiwan Ocean University, Keelung City 202, Taiwan; 10 Graduate Institute of Biomedical Engineering, National Chung Hsing University, Taichung City 402, Taiwan

**Keywords:** cord blood, microarray, inflammation, hematopoietic stem cells, CD38

## Abstract

To date, different experimental strategies have been developed for the *ex vivo* expansion of human hematopoietic stem cells (HSCs) for clinical applications. However, differences in the genomic function of expanded HSCs under different culture systems remain unclear. In this study, we compared the gene expression profiles of HSCs in *ex vivo* expanded serum (10% FBS, fetal bovine serum) and serum-free culture systems and analyzed the molecular functions of differentially expressed genes using microarray chips. We identified 839 differentially expressed genes between the two culture systems. These genes were enriched in the TNF -regulated inflammatory pathway in an FBS culture system. In addition, the mRNA expression of CCL2 (C-C motif chemokine ligand 2), TNF (tumor necrosis factor) and FOS (FBJ murine osteosarcoma viral oncogene homolog) was validated by RT-qPCR. Our data revealed that *ex vivo* expansion of HSCs using the FBS culture system induces an inflammatory response and high CD38 expression, indicating that this system might activate an inflammatory pathway and induce expression of the cancer marker CD38 during ex vivo expansion of HSCs. This study provides a transcriptional profile and new insights into the genomic functions of HSCs under different expanded cultures.

## INTRODUCTION

Hematopoiesis is the process of generating mature blood cells and immune cells, which originate in a few populations of hematopoietic stem cells (HSCs) [1, 2]. HSCs are defined as cells with both the capacity to self-renew and the ability to differentiate into various types of myeloid and lymphoid lineages [3, 4]. HSCs are frequently used in therapeutic applications to treat hematologic and immune disorders such as leukemia, anemia, congenital immunodeficiencies, metabolic disorders, and autoimmune diseases [5]. The CD34 antigen, an important biomarker that functions as a regulator of hematopoietic cell adhesion to stromal cells within the marrow microenvironment, is expressed on most HSCs. In clinical settings, cells expressing the CD34 antigen are considered HSCs, and the number of CD34^+^ cells infused proves to be the major prognostic factor for engraftment and survival [6]. In addition, many studies have demonstrated that the CD34^+^CD38^–^ fraction contains more primitive HSCs that can undergo hematopoiesis than the CD34^+^CD38^+^ fraction [7].

Currently, the main sources of HSCs for transplantation are collected from bone marrow, mobilized peripheral blood and cord blood (CB) [8]; however, the major limitation of CB HSC transplantation is the low number of HSCs in each CB unit, and this insufficient HSC number limits its treatment applications in children and delays the recovery of neutrophils and platelets after transplantation. Therefore, *ex vivo* expansion of CB HSCs can overcome the above limitations and is an important issue for clinical purposes [1].

For cell culture, serum, especially fetal bovine serum (FBS), is commonly used to support cell proliferation and cryopreservation, although the composition of each batch of serum varies [9]. Importantly, serum might contain bacterial, mycoplasma, and viral contamination and induce an immune response [10]. To comply with clinical regulations, the development of serum-free medium for HSC expansion is essential for basic research and clinical purposes.

**Figure 1:**
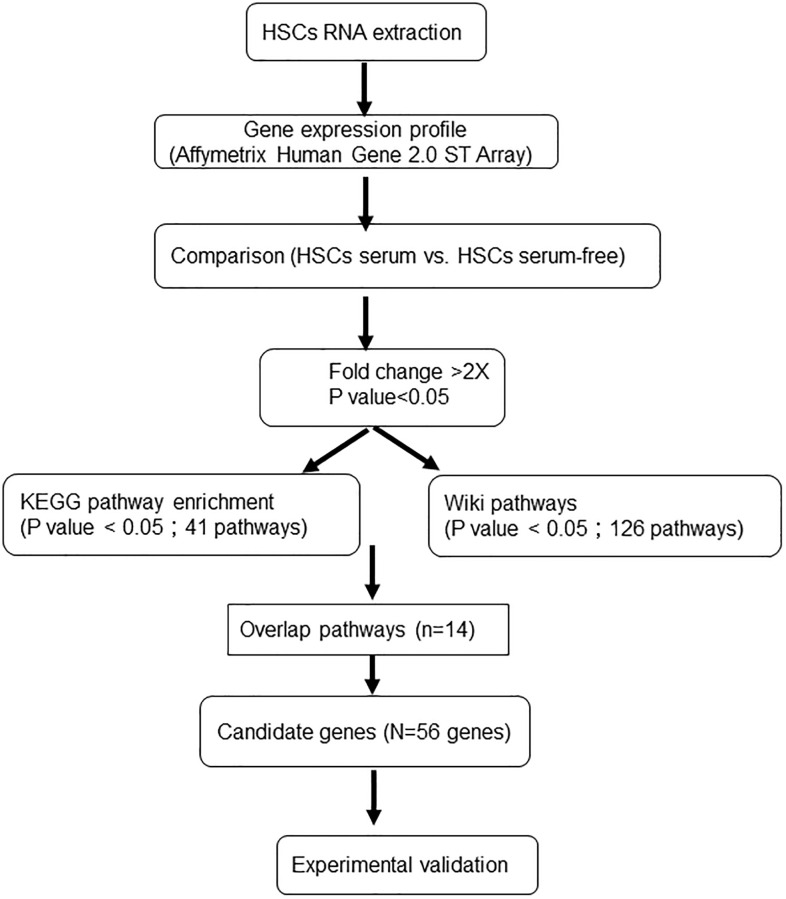
Experimental procedure for the gene microarray analysis of human HSCs expanded under different culture systems.

Previously, a serum-free medium for *ex vivo* expansion of HSCs (SF-HSC medium) was proposed by our study using systematic and statistical methodologies [11–13]. After one week of culture, the number of HSCs expanded over 30-fold and satisfied the criteria of colony-forming cells, long-term culture-initiating cells and severe combined immunodeficiency (SCID) mouse-repopulating cells. In addition, serum-free expanded HSCs maintained the ability to differentiate into various types of myeloid and lymphoid lineages *in vitro* [14–16]. Importantly, our results showed that serum-free expanded HSCs expressed high levels of CD34 and did not express CD38. It is worth noting that the HSCs cultured in SF-HSC medium plus 10% FBS also maintained CD34 expression but induced a high level of CD38 expression. The expression of CD38 in HSCs meant that the cells tended to differentiate and lose the characteristic of stemness. However, the mechanism and pathway between the FBS effect and CD38 expression for HSC expansion have not been well studied; therefore, we aimed to explore the key genes that caused the expression of CD38 in HSCs for expansion under serum-containing conditions using systematic biological approaches and then further investigated the effects of FBS on HSC expansion. In this study, we compared the gene expression profiles of *ex vivo-*expanded HSCs under serum-containing (10% FBS) and serum-free culture systems and then analyzed the molecular functions of differentially expressed genes using microarray chips (Figure 1). This study provides a transcriptional profile and new insights into the genomic functionality of HSCs under different expanded cultures. Taken together, we believe that serum-free expansion can provide a promising source of primitive and functional HSCs for basic research and clinical applications.

## RESULTS

### *In vitro* expansion of HSCs

**Figure 2:**
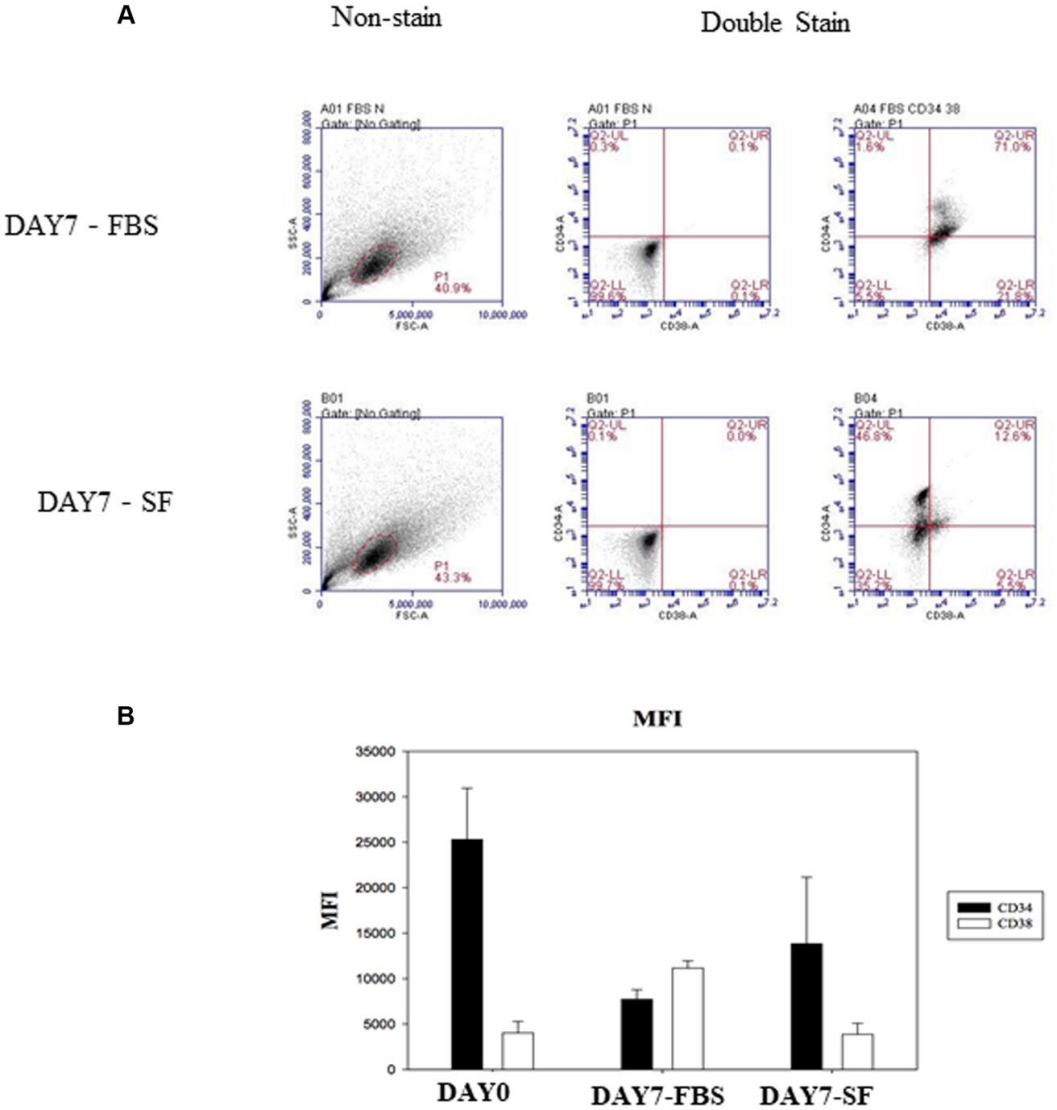
**Identify CD34 and CD38 expression by flow cytometry after HSCs cultured for 7 days.** (**A**) The isolated HSC (CD34^+^/CD38^–^) were examined by flow cytometry with SSC-conjugated anti-CD34 and FSC-conjugated anti-CD38. Non-stained HSCs are used to determine the background auto-fluorescence to set the negative population allowing cells stained with CD38 and CD34 to be visualized (right side). The percentage of surface marker-positive cells in the population is indicated. (**B**) Flow cytometry expression of CD34 and CD38 on hematopoietic stem cells. Comparison of mean fluorescence intensity (MFI) of CD34 and CD38 in serum-expanded (DAY7-FBS) vs. serum-free-expanded (DAY7-SF) HSCs.

We first assessed our two culture conditions as experimental systems for the *in vitro* expansion of human hematopoietic stem cells. A previous study indicated that specific serum factors stimulated CD38 expression in the erythroid developmental process [7]. To determine whether there were similar effects on undifferentiated HSCs, CD34 and CD38 expression was examined by flow cytometry. CD38 expression on 7-day-expanded HSCs of the serum culture system was increased compared to that on cells of the serum-free culture system (Figure 2). In addition, the expression of other surface antigens on HSCs was also demonstrated (Table 1).

**Table 1:** Hematopoietic stem cell surface markers expression under different culture systems.

**ID**	**Serum Avg (log2)**	**Serum-free Avg (log2)**	**Fold change**	***P*-val**	**Gene symbol**	**Description**	**Aliases**
16965268	7.7	5.47	4.67	4.01E-06	CD38	CD38 Molecule	
16698801	7.06	8.55	–2.81	1.53E-05	CD34	CD34 Molecule	
16974534	6.06	7.66	–3.03	1.48E-05	PROM1	Prominin 1	CD133
16903140	9.72	9.22	1.41	0.0029	CXCR4	Chemokine (C-X-C motif receptor 4)	
16745366	3.7	3.74	–1.03	0.8263	THY1	Thy-1 cell surface antigen	CD90
16966855	9.16	9.61	–1.36	0.0033	KIT	v-kit Hardy-Zuckerman 4 feline sarcoma viral oncogene homolog	c-Kit
16675578	9.47	8.62	1.8	0.0002	PTPRC	Protein tyrosine phosphatase, receptor type, C	CD45RA

### Global gene expression profiles of HSCs under different culture systems

**Figure 3:**
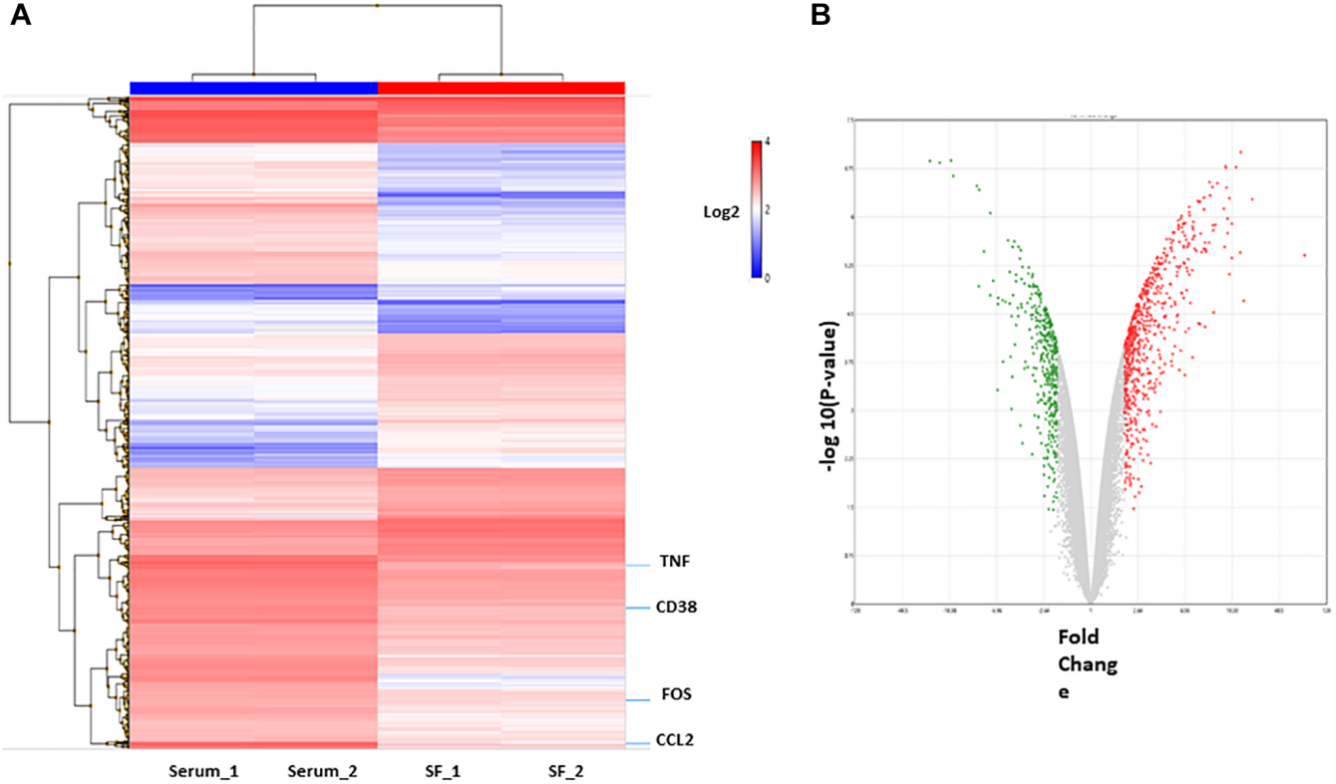
**Differentially expressed genes in culture system with or without serum.** (**A**) Hierarchical cluster showing the genes differentially expressed between the two groups. Significant genes (fold change of 2.0 and *p* < 0.05.) are shown in the heatmap. (**B**) The volcano plot shows 839 genes differentially expressed (578 genes upregulated (red) and 261 downregulated (green) in serum condition).

To identify the differences in the genomic functions of HSCs expanded *in vitro* under different culture systems, gene-expression profiles of *ex vivo* expanded serum and serum-free culture systems were compared, and then the molecular function of differentially expressed genes was analyzed (Supplementary Table 1). First, RNA was obtained from expanded HSCs and compared using cDNA microarrays. To define the differential expression profile within the different cell populations, Affymetrix Transcriptome Analysis Console (TAC) software was used. Genes with fold change >2 or <−2 and with *p*-value <0.05 were considered significantly altered between the conditions (HSC serum vs. HSC serum-free). In total, 1139 differentially expressed genes between the two culture systems were identified. Hierarchical clustering showed that the transcriptome profiles of the two cell populations were very different (Figure 3A). Of these, 578 genes were upregulated in the serum culture system, and 261 were downregulated (Figure 3B).

### Functional annotations of the differentially expressed genes

**Figure 4:**
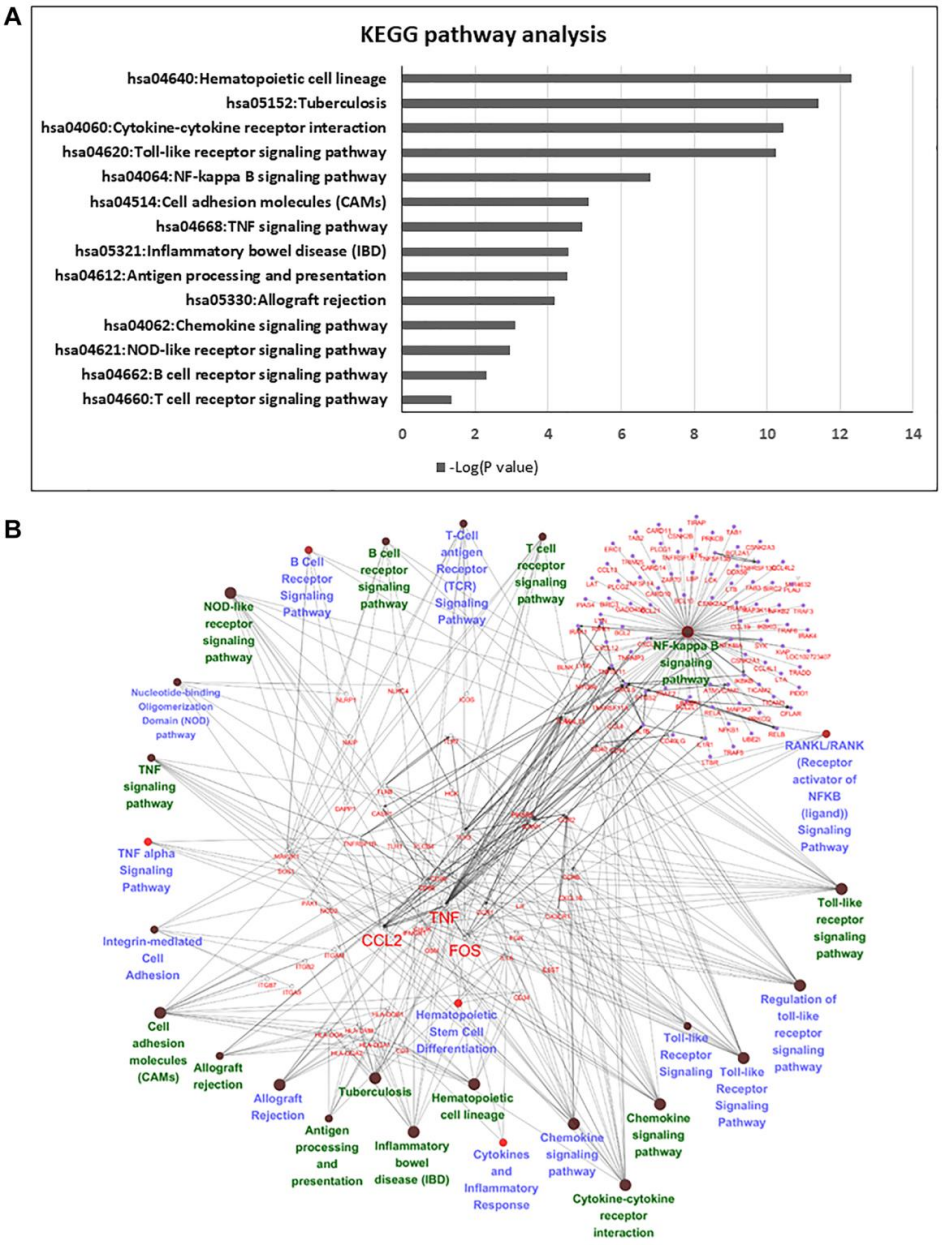
**Pathway analysis of differentially expressed genes in serum and serum-free culture systems.** (**A**) The list of candidate genes from 14 overlapping pathways between Wiki pathway and KEGG pathway. (**B**) Network analysis with cytoscape in 56 candidate genes in the 14 overlapping pathways.

Pathway enrichment analysis was then used to determine the biological roles of differentially expressed genes. Among these genes, we deleted no gene symbol genes that were no annotated in the databases, and 839 genes were chosen for further annotation. KEGG and Gene Ontology GO pathway analyses provided a measure of the critical functions that were preferentially represented by using DAVID software. Additionally, WikiPathways was also used to analyze the enriched pathways, and there were 14 overlapping pathways between KEGG and WikiPathways (Figure 4A), which included cytokine-cytokine receptor interaction, Toll-like receptor signaling pathway, NF-kappa B signaling pathway and TNF signaling pathway. Among these pathways, we found 56 candidate genes in our enrichment analysis. Most of these genes were classified as cytokines or chemokines that mainly regulated inflammation and the immune response of HSCs in the FBS culture system (Figure 4B). We listed significant differential expression genes involved in inflammation pathway in Supplementary Table 2.

### Confirmation of microarray analysis by experimental validation

**Figure 5:**
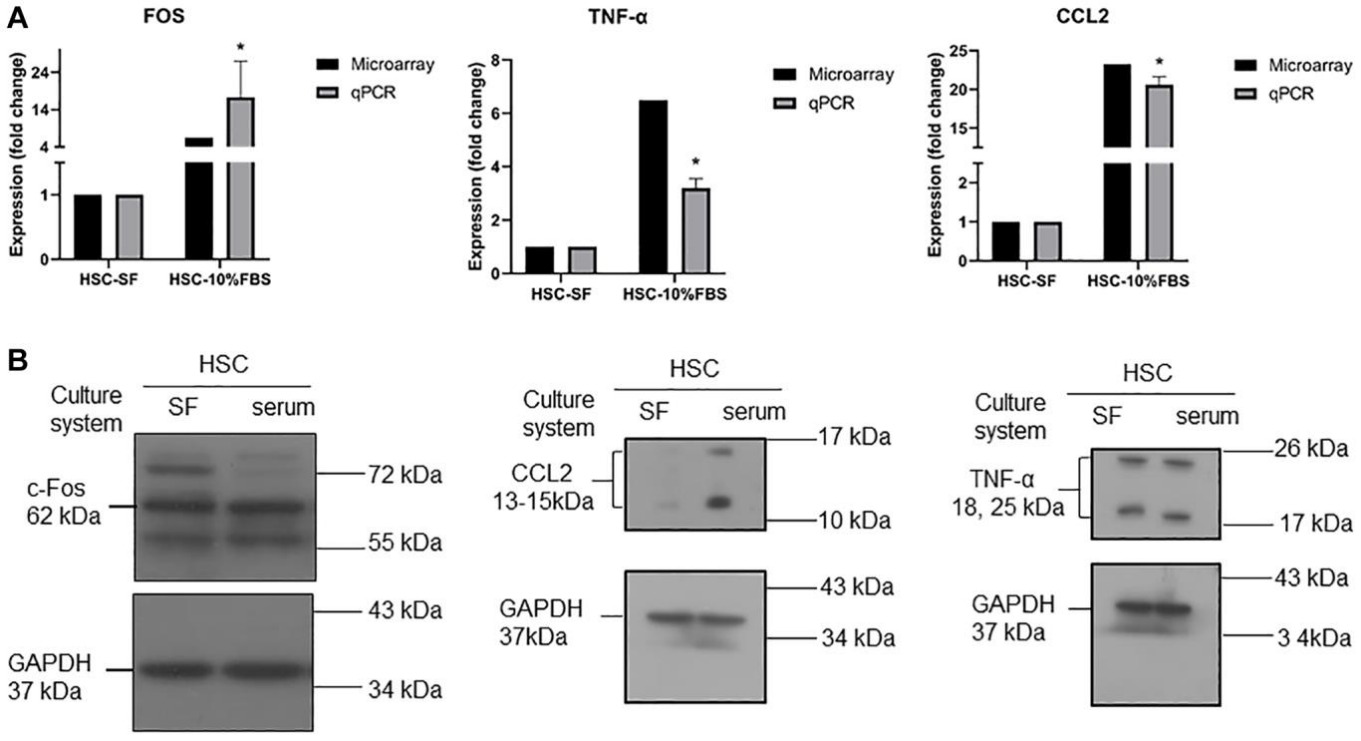
(**A**) RT-qPCR analysis for mRNA expression of CCL2, TNFα, and FOS in serum and serum-free cultured hematopoietic stem cells. Quantification of gene expression normalized with respect to GAPDH. (*n* = 3, data are expressed as means ± SD. ^*^p < 0.05 versus serum free condition). (**B**) Western blot analysis for protein expression of CCL2, TNFα, and FOS in serum and serum-free cultured hematopoietic stem cells. GADPH was used as a loading control.

To validate the microarray results, the CCL2, FOS and TNF genes were selected for experimental validation. These genes are mainly involved in the TNF signaling pathway, which plays an important role in regulating inflammation and cell survival or death [17]. We tested RT-qPCR and Western blotting to determine whether these genes were regulated at both the mRNA and protein levels, and as expected, the CCL2, FOS and TNF genes showed upregulated expression in serum-expanded HSCs (Figure 5A). At the protein level, we further confirmed that the CCL2 protein was increased in serum-expanded HSCs (Figure 5B), whereas FOS and TNF exhibited no significant change in protein expression. We further measured the protein levels in serum and serum-free cultured hematopoietic stem cells by ELISA kits. The results showed that the TNF-α protein levels were not significantly different between the two groups (Supplementary Figure 1).

### Expression of other blood cell CD markers on HSCs

**Figure 6:**
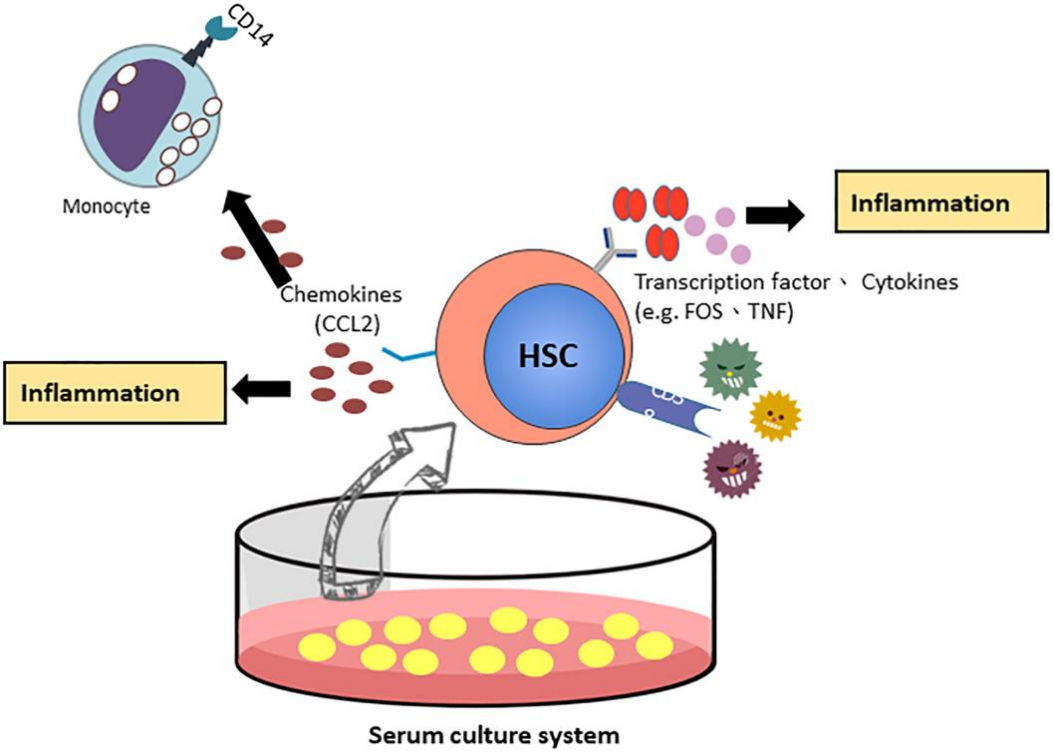
The proposed model of CCL2 involved HSCs *ex vivo* expanded culture systems.

By validation of RT-qPCR and Western blotting, CCL2 showed a similar pattern at the mRNA and protein levels. We focused on the CCL2 gene, which most likely regulates the function of HSCs in the FBS culture system. CCL2 is involved in the differentiation of hematopoietic stem cells into monocytes and regulates the inflammatory response of HSCs [18]. Next, we examined the expression of other mature blood cell CD markers in HSCs (Table 2). CD14 and CD4 were highly expressed in the FBS culture system, indicating that HSCs under serum culture conditions are more similar to differentiated blood cells and are associated with inflammation and immune function. CD38 is overexpression in inflammatory pathways [19] and CD38-deficient dendritic cells had an intrinsic inability to mobilize calcium and migrate in response to chemokines C-C chemokine ligand (CCL) [20, 21]. The Schematic showing the FBS serum increase CD38 expression through the C-C chemokine ligand (CCL) associated inflammation pathway (Figure 6).

**Table 2:** Other blood cells CD markers expression from HSCs microarray results.

**Monocyte/Macrophage**							
**CD marker**	**Gene symbol**	**ID**	**Serum Avg (log2)**	**Serum-free Avg (log2)**	**Fold change**	***P*-val**	**Description**
CD14^*^	CD14	17000793	7.17	3.88	9.8	0.00000343	CD14 Molecule
CD16	FCGR3A	16695700	2.62	2.66	–1.03	0.83	Fc fragment of IgG, low affinity llla, receptor (CD16a)
	FCGR3B	16695715	1.5	1.67	–1.3	0.5408	Fc fragment of IgG, low affinity lllb, receptor (CD16b)
**NK Cells (Natural killer cells)**							
**CD marker**	**Gene symbol**	**ID**	**Serum Avg (log2)**	**Serum-free Avg (log2)**	**Fold change**	***P*-val**	**Description**
CD56	NCAM1	16731297	2.31	2.43	–1.09	0.2209	Neutral cell adhesion molecule 1
**CD marker**	**Gene symbol**	**ID**	**Serum Avg (log2)**	**Serum-free Avg (log2)**	**Fold change**	***P*-val**	**Description**
**T Cells**							
CD3	CD3D	16745016	2.03	2.16	–1.1	0.336	CD3d molecule, delta (CD3-TCR complex)
	CD3E	16731795	2.65	2.51	1.11	0.16	CD3e molecule, epsilon (CD3-TCR complex)
	CD3G	16731806	2.54	2.53	1.01	0.9389	CD3g molecule, gamma (CD3-TCR complex)
CD4^*^	CD4	16747417	6.66	5.09	2.99	0.0000478	CD4 molecule
CD8	CD8A	16899928	3.92	3.74	1.13	0.1165	CD8a molecule

A systematic approach with gene expression analysis of HSC expansion under different culture systems demonstrated significant differences between FBS and serum-free culture conditions. The results showed that FBS increased the expression of inflammatory signaling pathways that might affect their proliferation, expansion, and differentiation patterns in culture. This finding was consistent with HSC biology and might have implications for HSC expansion and transplantation [22].

## DISCUSSION

In this study, CD34^+^ cells were isolated from human newborn UCB by the magnetic cell sorting method, and the collected HSCs were transferred into an *ex vivo* culture system under 2 different conditions (serum-free and serum-containing HSC media separately). We demonstrated that the serum-free expanded cells could preserve more primitive stemness, such as surface antigen expression of CD34^+^ and CD38^–^; however, the HSCs expanded in the serum-containing (FBS) system for 7 days might express the CD38 surface antigen. In general, the CD34^+^/CD38^–^ immunophenotype defines a primitive subpopulation of HSCs [23]. CD38 is a kind of NAD glycohydrolase that is usually expressed on erythrocytes, monocytes and macrophages but not on primitive HSCs [7]. In addition, some investigators found that CD38 was more active in the erythrocytes of patients with cancer and systemic diseases, and the increased CD38 expression was notably significant [24].

In normal physiology, the balance between HSC self-renewal and differentiation relies on HSC niches, which are a complex and dynamic environment [25–27]. Since HSCs have great potential for cell therapy, establishing a static *ex vivo* expansion system under homeostasis and mimicking the physiological HSC niche might be the key step for transplantation. HSCs from human newborn UCB master the continuous production of new blood cells throughout life and promise fast hematopoietic recovery after engraftment [28–30]. Previous studies have reported that HSCs could be expanded in the presence of serum [31, 32] or SF-HSC expansion systems with low and optimal cytokine concentrations [11, 33]. Hence, the aim of this study was to determine the discrepancy between the two culture systems via genomic tools.

According to the results of the microarray and pathway enrichment analyses, we found 14 overlapping pathways between KEGG and WikiPathways, including cytokine-cytokine receptor interaction, Toll-like receptor signaling pathway, NF-kappa B signaling pathway and TNF signaling pathway. Among these pathways, we chose three key genes related to inflammation (CCL2, c-FOS and TNF-α) from 56 candidate genes for further experiments. We focused on these three genes because they were mainly involved in the TNF signaling pathway and regulated cell inflammation, survival, and even death [17]. TNF signaling pathways are complex, and basically, the initial response to TNF is triggered by binding to TNFR1 (TNF receptor 1) and TNFR2 (TNF receptor 2) [34]. Both TNFR1 and TNFR2 have diverse functions and abilities in different tissues to interact with identical or unrelated molecules to regulate the downstream pathway [35]. To maintain homeostatic HSC stemness, cell-intrinsic and cell-extrinsic factors should be controlled tightly.

TNF-α (tumor necrosis factor-α) was named after the discovery of its cytotoxicity to tumor cells in 1984 [36]; it plays a kind of antineoplastic cytokine in cytotoxicity for promoting tumor development and progression [37]. In addition, TNF-α is also proinflammatory and involved in immunomodulation, inflammatory responses, and even all steps of leukemogenesis [34, 38, 39]. Of note, scientists have proven that TNF-α influences HSCs, especially HSCs with long-term exposure to proinflammatory cytokines, and might be linked to self-renewal abilities [40, 41]. HSCs possess both self-renewal and multipotent differentiation capacities; therefore, self-renewal maintenance without differentiation is a crucial issue for *ex vivo* HSC expansion systems [42].

CCL2 (also called monocyte chemoattractant protein-1, MCP-1) is the best-known CC chemokine, and the physiological functions of CCL2 induce a proinflammatory response on monocytes in the circulatory system [43]; moreover, CCL2 signaling is imperative to recruit mast cells to the tissue, indicating that CCL2 is involved in inflammatory cell trafficking [18]. Another interesting concept is c-FOS, which is a member of the FOS family of transcription factors and can cooperate with c-Jun as a heterodimer (activator protein-1, AP-1). Indeed, c-FOS is regarded as a proto-oncogene, and there have been many reports in the past related to FOS expression in human tumors [44]. Furthermore, it is well known that c-FOS signaling is hypersensitive to the addition of FBS [45, 46]. In other words, serum-containing conditions increased CCL2 expression during HSC expansion. These *ex vivo* serum-containing expanded cells might directly or indirectly interact with immune cells post engraftment, and thus, might contribute to the outcome of serious inflammation. However, the TNF and FOS protein expression results did not match the mRNA levels. The mRNA-protein relationship remains to be investigated and needs to be further validated. A previous study demonstrated that TNF gene expression is controlled at the transcriptional level in human monocytes [47]. Protein levels are largely determined by transcript concentrations, and posttranscriptional processes may lead to stronger deviations from an ideal correlation [48]. The protein and mRNA expression are determined by the relationships between the rates of the processes producing and degrading the participating molecules. Additionally, post-transcriptional, translational and degradation regulation affected the protein levels. The gene expression levels were not frequently reflected at the protein level and the correlation can be as little as 40% depending on the system [49, 50].

In conclusion, the SF-HSC expansion system mimics steady HSC niches for primitive stemness preservation, and it could provide a safe and high-quality cell source for HSC engraftment. To date, the FBS-based culture system still possesses too many unknown or unpredictable risks, but in contrast, the formula of SF-HSC medium is clearly defined, and the quality is totally guaranteed by standard procedures. As a consequence, we believe that this technique is promising and ready for clinical applications.

## MATERIALS AND METHODS

### Isolation of HSCs from umbilical cord blood

Newborn umbilical cord blood (UCB) was collected after approval from the Institutional Review Board of Taoyuan General Hospital, Ministry of Health and Welfare, Taiwan (TYGH104058). After obtaining donor consent, the isolation methods for CD34+ HSCs were as described previously. Briefly, mononuclear cells were isolated from UCB by Ficoll-Paque (Amersham Biosciences, Uppsala, Sweden) density gradient centrifugation. Freshly isolated CD34^+^ HSCs were obtained from mononuclear cells by magnetic cell sorting using CD34 microbeads and a VarioMACS Separator (Miltenyi Biotec Gmbh, Bergisch Gladbach, Germany).

### *Ex vivo* culture and expansion of HSCs

For HSC expansion under serum-free conditions, CD34^+^ cells were initially seeded at 5 × 10^4^ cells/mL in SF-HSC medium at 37°C in a 5% CO_2_ incubator for 7 days. SF-HSC medium was prepared with Iscove’s modified Dulbecco’s medium (IMDM) containing a cytokine cocktail (8.5 ng/mL thrombopoietin (TPO), 4.1 ng/mL IL-3, 15 ng/mL stem cell factor (SCF), 6.7 ng/mL Flt3-ligand, 0.8 ng/mL IL-6, 3.2 ng/mL G-CSF and 1.3 ng/mL GM-CSF; all from PeproTech, Inc., Rocky Hill, NJ) and serum substitutes (1.5 g/L human serum albumin, 4.4 mg/mL human insulin, 60 mg/mL iron-saturated human transferrin and 25.9 μM 2-mercaptoethanol; all from Sigma-Aldrich, St Louis, MO, USA) and sterility and lack of mycoplasma contamination was confirmed. For HSC expansion under serum-containing conditions, CD34^+^ cells were initially seeded at 5 × 10^4^ cells/mL in SF-HSC medium plus 10% fetal bovine serum (FBS, GE Healthcare Bio-Sciences, Pittsburgh, PA) at 37°C in a 5% CO_2_ incubator for 7 days. After a 7-day expansion culture, the characteristics of serum-free expanded HSCs were compared with those of serum-containing expanded HSCs (Figure 1).

### Analysis of surface antigens

To analyze the surface antigens, anti-CD34 and anti-CD38 fluorescence monoclonal antibodies (eBioscience, San Diego, CA, USA) were used. Matched labeled isotypes were used as controls. The labeled cells were analyzed using a BD Accuri™ C6 flow cytometer with CFlow Plus software (Becton-Dickinson Biosciences, San Jose, CA, USA).

### RNA extraction

HSCs were expanded as described above from the same batch of cord blood donations in a 6-well plate. After 7 days of culture, high-quality total RNA was extracted and purified from HSCs grown in different culture systems using the RNeasy Mini Kit (Qiagen, Hilden, Germany). All procedures were performed according to the manufacturers’ instructions.

### Microarrays and gene expression analysis

The quality of the RNA samples was measured in an Agilent 2100 Bioanalyzer (Agilent Technologies, Foster City, CA, USA). RNA integrity numbers (RINs) greater than 8 were further processed for microarray studies. Gene expression profiles were evaluated in the Affymetrix GeneChip™ Human Gene 2.0 ST Array (Thermo Fisher Scientific, CA, USA). The detailed protocol for fragmentation, hybridization, washing, staining, and further processing of the arrays was performed according to the manufacturer’s protocol. Array data analysis at the gene level was conducted with Affymetrix Transcriptome Analysis Console (TAC) software. Genes with fold change >2 or <−2 and with *p*-value < 0.05 were considered significantly differentially expressed between the conditions (HSCs serum vs. HSCs serum-free). All data are publicly available in the Gene Expression Omnibus database (GEO ID: GSE126909).

### Functional annotation and pathway analysis

Functional annotation in differentially expressed gene sets was assessed using the Database for Annotation, Visualization, and Integrated Discovery (DAVID) tool (http://david.abcc.ncifcrf.gov/) and WIkiPathways. Gene Ontology (GO) and Kyoto Encyclopedia of Genes and Genomes (KEGG) analyses from the DAVID [51, 52] tool displayed annotated genes involved in enriched pathways if the calculated Expression Analysis Systematic Explorer (EASE) score was below 0.05. Integration of biological networks was performed using Cytoscape [53].

### Real time RT-PCR

To confirm differentially expressed genes identified by the microarray analysis, quantitative real-time polymerase chain reaction (RT-qPCR) was performed for the following three selected genes: FOS, CCL2 and TNF. First, a total of 0.1 μg of RNA was converted to first-strand cDNA using the ProtoScript^®^ II First Strand cDNA Synthesis Kit (New England Biolabs, Inc., USA) in the presence of oligo-dT primers. After cDNA amplification reactions were run using the ABI™ StepOne™ Real-Time PCR System (Applied Biosystems, Foster City, CA, USA) with KAPA SYBR^®^ FAST Master Mix (2X) ABI Prism™ (KAPA BIOSYSTEMS, Boston, MA, USA), the gene expression levels were normalized to the expression level of the internal housekeeping gene GAPDH. Relative quantification was calculated using the 2^−ΔΔCT^ method. All oligonucleotides used in this study were designed with PRIMER 3 software available online (http://bioinfo.ut.ee/primer3-0.4.0/). The sequences of the forward (F) and reverse (R) primers are shown in Supplementary Table 3. The results were normalized to GAPDH quantified from the same samples.

### Western blotting

For protein extraction, the cells were harvested from the culture dishes and washed using PBS before lysing homogeneously in 30 μl PRO-PREP™ Protein Extraction Solution (iNtRON Biotechnology). Thirty micrograms of total protein were dissolved in 5X loading dye, heated at 95°C for 10 min, and then placed on ice for 3 min for centrifugation. The proteins were separated using SDS-PAGE (5% stacking gel/10 and 12% separating gel) and transferred to a polyvinylidene difluoride (PVDF) membrane. Then, the membrane was blocked using 5% milk in TBST for 1 hour and incubated with a primary antibody (at the appropriate dilution and diluent as recommended in the product datasheet) with gentle agitation overnight at 4°C. The antibodies were purchased as follows: anti-GAPDH antibody [GT239] (HRP) (GeneTex, Inc., USA), rabbit anti-c-Fos polyclonal antibody (Santa Cruz Biotechnology, Santa Cruz, CA), rabbit anti-CCL2 polyclonal antibody (#2027) (Cell Signaling Technology, Leiden, The Netherlands), and anti-TNF-α (D5G9) rabbit monoclonal antibody (Cell Signaling Technology, Leiden, The Netherlands). The membrane was then incubated for 1 hour at RT with anti-rabbit HRP-conjugated secondary antibodies to detect the labeled bands using the Pierce™ ECL substrate (Thermo Fisher Scientific, CA, USA).

### TNFα ELISA

The cell-free supernatants were used to determine TNF-α in the conditioned medium with a Human TNF-alpha DuoSet ELISA kit (R&D Systems, Inc., USA).

## Supplementary Material

Supplementary Figure 1

Supplementary Table 1

Supplementary Tables 2 and 3
