# Supplementary material for: CCL2 associated with CD38 expression during ex vivo expansion in human cord blood-derived hematopoietic stem cells
Source: Aging (Albany NY). 2021 Aug 10;13(15):19878–93. doi: 10.18632/aging.203398 (PMC8386547; doi:10.18632/aging.203398)
Supplement: Supplementary Figure 1 [file aging-13-203398-s001.pdf]

SUPPLEMENTARY FIGURE

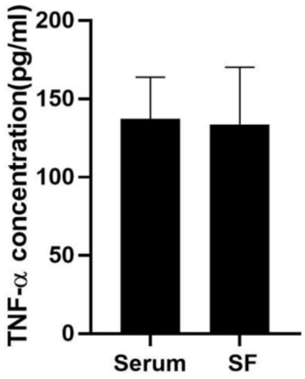

Supplementary Figure 1. Protein levels in serum and serum-free cultured hematopoietic stem cells by ELISA kit.
