# Supplementary material for: CCL2 associated with CD38 expression during ex vivo expansion in human cord blood-derived hematopoietic stem cells
Source: Aging (Albany NY). 2021 Aug 10;13(15):19878–93. doi: 10.18632/aging.203398 (PMC8386547; doi:10.18632/aging.203398)
Supplement: Supplementary Tables 2 and 3 [file aging-13-203398-s003.pdf]

SUPPLEMENTARY TABLES

Please browse Full Text version to see the data of Supplementary Table 1.

Supplementary Table 1. List of differential expressed genes in HSCs with two culture systems.

Supplementary Table 2. List of significant differential expression genes involved in inflammation pathway.

ID	fbs Avg (log2)	sf Avg (log2)	Fold Change	*P*-val	FDR *P*-val	Gene Symbol
16940172	8.35	4.83	11.49	2.83E-07	0.0013	CCR2
16711501	6.83	2.81	16.27	3.46E-07	0.0013	IL2RA
16952874	7.54	3.42	17.31	5.05E-07	0.0013	CCR1
17106493	8.59	6.15	5.43	1.72E-06	0.0016	IL13RA1
16843602	7.39	4.6	6.91	1.93E-06	0.0017	CCL3L3; CCL3L1
16833426	6.14	3.39	6.74	2.76E-06	0.0018	CCL4L2; CCL4L1
16989496	6.66	4.76	3.73	9.84E-06	0.0029	TGFBI
17024144	6.82	5.22	3.02	1.09E-05	0.003	IFNGR1
16950216	4.99	3.4	3.01	1.33E-05	0.0033	IL5RA
17006659	8.71	6.01	6.51	1.52E-05	0.0033	TNF
16833204	9.83	5.29	23.34	1.97E-05	0.0038	CCL2
17116694	5.48	7.05	–2.96	3.13E-05	0.0047	IL9R
16933760	6.46	4.8	3.17	4.82E-05	0.0057	LIF
16908305	6.83	3.44	10.51	5.02E-05	0.0057	CXCR2P1
16883715	5.53	4.08	2.73	5.23E-05	0.0058	IL18R1
16786587	6.13	5.05	2.11	0.0002	0.0105	FOS
16833420	3.66	2.6	2.08	0.0011	0.0274	CCL4
16840113	5.05	3.82	2.36	0.0015	0.0325	CXCL16

Supplementary Table 3. Primer sequences for real time quantitative RT-PCR.

Name	Sequence (5′–3′)	Size (mer.)
GAPDH-F	TGCACCACCAACTGCTTAGC	20
GAPDH-R	GGCATGGACTGTGGTCATGAG	21
FOS-F	AGAATCCGAAGGGAAAGGAA	20
FOS-R	AGGGCCCTTATGCTCAATCT	20
CCL2-F	GCTCATAGCAGCCACCTTCATTC	23
CCL2-R	GGACACTTGCTGCTGGTGATTC	22
TNF-α-F	CCAGGCAGTCAGATCATCTTC	21
TNF-α-R	GTTATCTCTCAGCTCCACGC	20
